# Report of the Autopsy on the Central Park Chimpanzee

**Published:** 1890-04

**Authors:** Jo H. Linsley

**Affiliations:** Pathologist to the New York Post-Graduate Hospital and New York Infant Asylum; Lecturer on Pathology in the Medical Department, University of Vermont; 41 East 21st Street, N. Y.


### REPORT OF THE AUTOPSY ON THE CENTRAL PARK CHIMPANZEE.

By Dr. Jo H. Linsley, M.D.,
*Pathologist to the New York Post-Graduate Hospital and New York Infant Asylum; Lecturer on Pathology in the Medical Department, University of Vermont.*

The subject of this notice was known as Kitty O'Brien. Briefly, her history is as follows: She was received at the Park

July 12th, 1887, and at that time was supposed to be about 2 years old. She continued in good health until about August, 1889, when on account of annoyance overhead produced by the swinging of the trapeze, the trapeze was tied up. In October, 1889, she began to grow listless, and had a distaste for food. Her lungs were supposed to be affected, and she was given an emulsion of cod liver oil. From that time on she gradually declined, and died March 1st, 1890, about 4 A. M.

Autopsy, held at the Museum of Natural History, March 1st, 1890, at 2.45 P. M.:

INSPECTION.—There is considerable œdema and serous infiltration of the cellular tissue, especially about the abdomen. There is a fair amount of subcutaneous fat.

PERITONEUM.—Appears normal. The peritoneal cavity contains 2000 cc. of yellow serum.

DIAPHRAGM.—On the left side, at the sixth space; on the right, at the seventh rib.

THORAX.—The left pleural cavity contains about 1000 cc., and the right pleural cavity, 500 cc., of clear yellow serum. There are no adhesions on the left side; there are adhesions on the right side posteriorly, for a short distance.

HEART.—Weighs 150 grm. The two surfaces of the pericardium are firmly adherent throughout the whole extent of this structure; the valves are competent. Between the arch of the aorta and the bronchi, and firmly attached to both, is a cheesy tubercular mass, 4 ctm. in diameter.

BRONCHIAL GLANDS.—Several bronchial glands are somewhat enlarged and congested; but only the one above described shows apparently tubercular degeneration.

LUNGS.—Left weighs 100 grm.; poorly aerated, and somewhat œdematous; otherwise normal. Right weighs 100 grm.; is completely collapsed—sinks in water. One small cheesy mass about 1 ctm. in diameter is found near the apex.

SPLEEN.—Weighs 90 grm. Two small cheesy nodules are seen; otherwise the organ appears normal.

KIDNEYS.—Left weighs 120 grm.; somewhat congested; the markings are not distinct; the capsule is not adherent. Right weighs 120 grm. Both kidneys are alike.

INTESTINES.—Appear normal.

Mesenteric Glands.—Are normal in appearance.

Stomach.—The mucous membrane shows numerous punctate hemorrhages or points of congestion. It contains quite a considerable quantity of partly digested food.

Liver.—Weighs 810 grm.; it is congested in part. On section, it presents areas, some of which are irregular, and others fairly round, and varying in size from a pin's head to 4 ctm. in diameter. They are pale and of an even, soft consistency, nearly similar to that of the adjacent nearly normal appearing liver tissue. These pale areas constitute more than one-half of the entire organ. The other portions of the liver are dark-brown. There are no cheesy nodules. The orifice of the common bile duct is pervious. The gall bladder is apparently normal.

Pancreas.—Weighs 50 grm., and is apparently normal.

Bladder, Uterus and Ovaries, *combined.*—Weigh 80 grm. They are normal in appearance.

### Microscopical Examination.

Heart.—The muscular tissue shows much small round-cell infiltration. Narrow areas are seen, which to the unaided eye have the appearance and consistence of calcareous deposit, and which under the microscope have an even, homogeneous, granular appearance. This substance presented great obstacles to cutting sufficiently thin sections.

Pericardium.—Sections through the pericardium, and including some of the muscular tissue of the heart, showed complete infiltration of the latter with small and large round cells. The pericardium itself is the seat of innumerable inflammatory corpuscles. Many fibres, apparently fibrin, are seen. Between the muscular tissue and the free surface of the pericardium there is a large deposit of amorphous looking matter, supposed to be calcareous.

Bronchial Glands.—Upon attempting to make sections of the gland, its centre was found to be so broken down as to make it impossible for it to keep its shape for mounting. The entire centre of the gland was exceedingly soft. It was not found practicable to make a microscopical examination of this tissue.

Lungs.—The alveolar walls are thickened and infiltrated with inflammatory corpuscles; the walls of the small bronchi are thickened and infiltrated with these same corpuscles. The pleura is thickened and contains small round cells in great abundance. These same cells are seen in large numbers in the lumen of the bronchi; also granular matter, and epithelial cells in a degenerative condition; some well-defined delicate fibres, apparently of fibrin. The walls of the bronchi seem to be somewhat collapsed; the bloodvessels are congested, and many of the smallest bronchi are open, but nearly all the air vesicles appear either collapsed or filled with granular matter including epithelium, small round cells, and a little blood, or both. The walls of the bloodvessels are thickened. A very small portion of the lung examined shows a fair amount of distention of the air vesicles, and an absence of matter in their interior. In the greater part of the organ the alveolar walls are ill defined. No areas resembling tubercular degeneration are seen.

SPLEEN.—The capsule is very greatly thickened; there is an increase of the fibrous connective stroma of the organ; the walls of the bloodvessels are also thickened; no tubercular nodules are seen.

KIDNEYS.—The fibrous tissue of the kidney is increased; the capsules of Bowman are thickened, and immediately beneath a few of them are seen many blood corpuscles. The Malpighian bodies completely filled up the capsules. The epithelium lining the urinary tubules is very granular, and in many places has desquamated. The tubules are filled with granular matter. Many small round cells are everywhere seen. There is some fatty and some amyloid degeneration in a few of the glomeruli. A few vessels are seen filled with blood; but the greater number are not.

INTESTINES.—In places the epithelial tissue shows a hyperplasia, and the villi are indistinguishable, and there is very great infiltration of inflammatory corpuscles in their location. The morbid process does not appear to extend beneath the submucosa.

LIVER.—The liver contains a great excess of lymphoid cells; extensive fatty infiltration and degeneration are seen. There is a considerable increase of fibrous stroma; the liver cells are exceedingly granular; and throughout a great portion of the gland they are seen to be either nearly or completely replaced by granular matter. The capsule of Glisson is very greatly thickened, and immediately beneath it, in places, is seen a hemorrhage, the blood extending into the liver tissue.

The case is a most interesting one from a pathological standpoint. While it is impossible to absolutely demonstrate the primary source of infection, still I am convinced that this must have been in the bronchial gland found closely adherent to the aorta and trachea. My reason for it is, principally the extensive and apparently old tubercular degeneration found in it. In this particular, the change was very much more strongly marked than in any nodule found elsewhere in the body. The tubercular process evidently extended from the gland to the heart, and produced a tubercular pericarditis. The involvement of the heart so seriously affected the general circulation as to produce anasarca and pleuritic effusions. There are strong evidences of there being portal phlebitis, the portal vein showing indications of inflammatory action. This would easily account for the peculiar appearance of the liver, whose circulation was the most seriously affected. The periphery of the acini were particularly anæmic, while their centres were more or less congested. There has been no satisfactory staining of the sections of the bronchial gland for tubercle bacilli, it being absolutely impossible, up the present time, to procure sections thin enough to demonstrate these organisms even if present. I shall attempt to soften the tissues by chemical means, and if successful in obtaining sufficiently thin sections, and I find tubercle bacilli present, I shall report it to this journal. I am perfectly well satisfied, however, of the nature of the morbid process. Another point of interest is the fact that while the animal was supposed to be suffering from pulmonary tuberculosis, still the only sign of tubercle found in either lung was the small cheesy mass situated in the apex of the right lung, as before described. This was not extensive enough to give rise to any marked pulmonary symptom. The brain was removed by Dr. N. E. Brill for Dr. E. C. Spitzka, and I have not yet learned the result of his investigation.

41 East 21st Street, N. Y.

